# Spectral Imaging Techniques in Heart Disease Assessment Using Photon‐Counting Detector Computed Tomography

**DOI:** 10.1111/echo.70177

**Published:** 2025-08-18

**Authors:** Sámuel Beke, Emese Zsarnóczay, Lili Száraz, Borbála Vattay, Dmitrij Kravchenko, Milan Vecsey‐Nagy, Giuseppe Tremamunno, Tilman Emrich, Akos Varga‐Szemes, Bálint Szilveszter, Pál Maurovich‐Horvat

**Affiliations:** ^1^ Department of Radiology, Medical Imaging Centre Semmelweis University Budapest Hungary; ^2^ Heart and Vascular Center Semmelweis University Budapest Hungary; ^3^ Department of Radiology and Radiological Science Medical University of South Carolina Charleston USA; ^4^ Department of Diagnostic and Interventional Radiology University Hospital Bonn Bonn Germany; ^5^ Department of Medical Surgical Sciences and Translational Medicine Sapienza University of Rome ‐ Radiology Unit – Sant'Andrea University Hospital Rome Italy; ^6^ Department of Diagnostic and Interventional Radiology University Medical Center of the Johannes Gutenberg‐University Mainz Germany; ^7^ German Centre for Cardiovascular Research Partner site Rhine‐Main Mainz Germany

**Keywords:** material decomposition, photon‐counting detector computed tomography, spectral imaging, virtual monoenergetic images

## Abstract

The photon‐counting detector (PCD) system was developed to overcome the limitations of traditional energy‐integrating detectors (EIDs) in computed tomography. This technique results in a higher contrast‐to‐noise ratio (CNR), better spatial resolution, and additionally provides detector‐based spectral information. Spectral imaging is based on the acquisition and detection of different photon energy spectra. By using spectral information, image quality (IQ) can be improved, and materials can be distinguished; furthermore, their quantity and concentration can be determined. Spectral data‐based parameters and reconstructions can be utilized in calcification measurement, luminal stenosis assessment, and myocardium characterization. The aim of this review is to summarize the spectral imaging applications of PCD computed tomography in the assessment of heart diseases.

## Graphical abstract

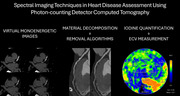

**Abbreviations**
AVC: aortic valve calcification
CAC: coronary artery calcium
CACS: coronary artery calcium score
CCTA: coronary CT angiography
CMR: cardiac magnetic resonance imaging
CNR: contrast‐to‐noise ratio
EAT: epicardial adipose tissue
ECV: extracellular volume
EID: energy‐integrating detector
FAI: fat attenuation index
ICA: invasive coronary angiography
IQ: image quality
LE: late enhancement
MAC: mitral annular calcification
MPI: myocardial perfusion imaging
PCD: photon‐counting detector
QIR: quantum iterative reconstruction
TAVR: transcatheter aortic valve replacement
TNC: true non‐contrast
UHR: ultra‐high resolution
VMI: virtual monoenergetic image
VNC: virtual non‐contrast
VNCa: virtual non‐calcium
VNI: virtual non‐iodine

## 1 Introduction

The photon‐counting detector (PCD) system was developed to overcome the limitations (e.g., insufficient spatial resolution, artifacts of extensive calcification, and metallic objects) of traditional energy‐integrating detectors (EID) in computed tomography (CT). PCDs convert individual x‐ray photons directly into an electric signal while EIDs require an additional step (scintillation) of converting photons into an electric signal. In PCDs, each photon generates an electrical pulse with a height proportional to the energy the photon deposits. The detector counts the number of pulses with heights that exceed the preset threshold level and sorts the incoming photons into energy bins based on their energy. This technique results in a higher contrast‐to‐noise ratio (CNR) and better spatial resolution, also providing detector‐based spectral information. The technical performance of PCD‐CT is improved relative to current EID‐CT systems [1, 2, 3, 4]. The aim of this review is to summarize the spectral imaging applications of PCD‐CT in the assessment of heart diseases.

### 1.1 Spectral Imaging

Spectral imaging is based on the acquisition and detection of different photon spectra. In dual‐energy CT, one high‐energy and one low‐energy x‐ray photon spectrum are detected. Using PCD with several energy bins, more than two energy levels can be separated. Attenuation is x‐ray photon energy dependent; each voxel will have a different Hounsfield unit (HU) value on different energy level acquisitions. The attenuation characteristics of basic materials (calcium, water) can be expressed as a function of HU at low and high photon energies. Elements with high atomic numbers show a step change in the attenuation at a specific x‐ray energy (at the binding energy of the inner electronic K‐shell), called K‐edge, that identifies the corresponding element uniquely. These materials, therefore, can be distinguished, and their concentration can be determined. This process is called material decomposition and enables virtual image reconstruction to create images without the depiction of one or two materials (e.g., virtual non‐calcium [VNCa] or virtual non‐iodine [VNI] images) [1, 5, 6, 7].

Virtual monochromatic (or monoenergetic) images (VMI) simulate the appearance of images obtained with a monochromatic x‐ray source. They are generated by a process equivalent to material decomposition. These images improve CNR by using low‐energy level (measured in keV) reconstructions, enabling lower contrast agent volume, and reducing image artifacts (beam‐hardening, blooming) with high‐energy level reconstructions [1, 5, 8, 9].

## 2 Calcium Scoring

The Agatston score of coronary artery calcium (CAC) is based on the calcified plaque area and the maximal density of individual calcified lesions, acquired using a 120 kVp acquisition scan with a slice thickness of 2.5–3 mm [10, 11, 12]. In the following chapters, CACS (coronary artery calcium score) will refer to the Agatston score. PCD‐CT is accurate in CACS measurement compared to EID‐CT, proven by phantom, ex vivo, and in vivo studies [13, 14, 15, 16]. CAC measurement can be more precise with PCD‐CT due to its better spatial resolution, reduced blooming artifacts, and utilization of spectral data.

### 2.1 VMIs in Calcium Scoring

**FIGURE 1:**
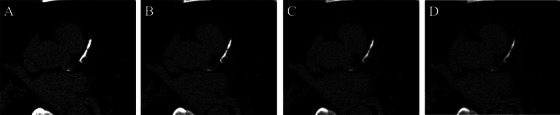
Different VMI reconstructions of calcium scoring images. Non‐contrast calcium scoring scan (Siemens NAEOTOM Alpha) of a 68‐year‐old male patient. A, B, C, and D show virtual monoenergetic reconstructions at 50, 70, 90, and 120 keV, respectively, both reconstructed with Qr36 kernel, QIR off, and 3 mm slice thickness. CACS was calculated with syngo.via Calcium Scoring (Siemens Healthineers) at each keV. 50 keV CACS = 1035; 70 keV CACS = 927; 90 keV CACS = 705; 120 keV CACS = 541.

VMIs can be generated based on spectral information to enhance image contrast and reduce artifacts. Scanning with lower tube voltage (kV‐independent CAC scoring) can reduce radiation dose using VMI reconstructions [17]. Eberhard et al. [14] performed 120 kVp (120 kV peak tube voltage) scans on an anthropomorphic phantom and 20 patients. The phantom was scanned with different image quality (IQ) levels (20–80), which represents quality reference tube current time product (measured in mAs), that provides a system and reconstruction‐independent IQ definition. Patients were scanned with IQ level 20. Polychromatic (T3D) and VMI reconstructions were made at different keV (60–75) levels with QIR (quantum iterative reconstruction) off and all QIR levels (1–4) with 3 mm slice thickness. CACS was not affected by IQ level. The highest CACS were found at 60 keV, with significantly decreasing scores at each 5 keV increase. For all VMI, CACS were the highest with QIR off and significantly decreased at each increasing level of QIR. The most accurate CACS was achieved using VMI at 70 keV with QIR off or at 65 keV with QIR 3–4. An example of different VMI reconstructions of calcium scoring images is presented in Figure 1.

CACS evaluation at different tube voltages to reduce radiation dose was investigated in other studies. A dynamic phantom was scanned by van der Werf et al. [18] with five different protocols. VMI reconstructions were made at 70 keV. CACS using 90 kVp with 75% and 45% dose acquisitions were comparable with the 120 kVp standard acquisition for medium‐ and high‐density calcifications. However, low‐density calcifications were difficult to detect with a reduced dose. Mergen et al. [15] scanned an anthropomorphic phantom with extension rings to simulate different patient sizes at different tube and with EID‐CT as a reference. VMI reconstructions were performed at 65–70 keV with QIR off and QIR 1–4. Imaging at 90 kVp or tin‐filtered 100 kVp was associated with a size‐dependent radiation dose reduction between 23% and 48% compared to 120 kVp. Reconstructions with 65 keV, QIR 3 at 90 kVp, and 70 keV, QIR 1 at tin‐filtered 100 kVp allowed them to calculate CACS with a deviation of ≤5% for all phantom sizes, compared to the EID‐CT reference. In a dynamic phantom study, Dobrolinska et al. [19] showed that a tube voltage of tin‐filtered 100 kVp reconstructed at 70 keV enables a ≥19% dose reduction compared to 120 kVp, independent of phantom size, CAC density, and heart rate. In a dynamic phantom study, van der Werf et al. [20] determined monoenergetic level‐specific CACS thresholds, based on CAC comparison with the reference at full dose, 70 keV reconstruction. VMI level–specific CACS thresholds resulted in similar CACS as in standard reconstructions, with a potential dose reduction of 50%. The studies regarding dose reduction with tube voltage and tube current modulation are shown in Table 1.

**TABLE 1:** Summary of the studies regarding the effect of tube voltage and tube current modulation.

Study	Population	Acquisition	Reconstruction	Results
van der Werf et al. [18]	Dynamic phantom	120 kVp (ref.) Sn100 kVp 90 kVp 90 kVp (75% dose) 90 kVp (45% dose) all with IQ level 16	70 keV QIR off Qr36 3 mm slice thickness	90 kVp with dose reduction was comparable to ref. low‐density calcifications were difficult to detect
Mergen et al. [15]	Phantom with different sizes	120 kVp EID‐CT (ref.) 90 kVp Sn100 kVp 120 kVp Sn140 kVp all with IQ level 20	65 and 70 keV QIR off and 1–4 Qr36 3 mm slice thickness	Most accurate reconstructions: 65 keV, QIR 3 at 90 kVp (23% dose reduction) 70 keV, QIR 1 at Sn100 kVp (48% dose reduction)
Dobrolinska et al. [19]	Dynamic phantom with different sizes	120 kVp (ref.) 70 kVp 90 kVp Sn100 kVp Sn140 kVp 140 kVp all with IQ level 16	70 keV QIR off Qr36 3 mm slice thickness	70 keV at Sn100 kVp was accurate with 19% dose reduction
van der Werf et al. [20]	Dynamic phantom	120 kVp with 20 mAs (IQ level 16) 10 mAs	40–190 keV QIR off Qr36 3 mm slice thickness	70 keV at 120 kVp with 50% dose reduction was accurate for medium‐ and high‐density calcification

Abbreviations: IQ level, image quality level; keV, kiloelectronvolts; kVp, peak tube voltage in kV; QIR, quantum iterative reconstruction; Sn, tin‐filtered.

### 2.2 Quantify Calcium With Material Decomposition

Virtual non‐contrast (VNC) and VNI images can be reconstructed from spectral data of contrast‐enhanced scans applying material decomposition with dedicated algorithms, and CACS can be determined (which are usually marked as CACS_VNC_ and CACS_VNI_). The VNI images are created with the PureCalcium reconstruction, but in some publications, authors refer to them as VNC as well. This method could reduce scan time and radiation dose by eliminating unenhanced scans which are generally used for CAC scoring (true non‐contrast, TNC). Determining the CACS was feasible using VNC images obtained on dual‐layer spectral EID‐CT [21, 22].

**FIGURE 2:**
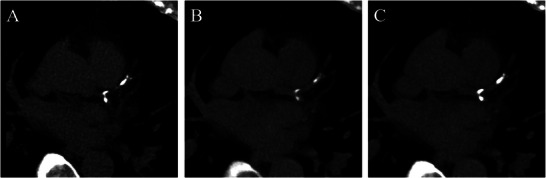
Comparison of TNC, VNC, and VNI reconstructions. (A) 70 keV VMI reconstruction of a true non‐contrast scan (TNC, Siemens NAEOTOM Alpha) of a 64‐year‐old male patient. (B) virtual non‐contrast (VNC) reconstruction, (C) 70 keV VMI virtual non‐iodine (VNI, PureCalcium) reconstruction of a contrast‐enhanced scan (Siemens NAEOTOM Alpha) of the same 64‐year‐old male patient. Both reconstructed with Qr36 kernel, QIR off, and 3 mm slice thickness. CACS was calculated with syngo.via Calcium Scoring (Siemens Healthineers) for each reconstruction. Total true non‐contrast CACS = 341; total virtual non‐contrast CACS = 142; total virtual non‐iodine CACS = 370.

First, Emrich et al. [23] compared CACSs measured based on VNI, VNC, and TNC images of an anthropomorphic phantom and 67 patients. All reconstructions were made at 70 keV, QIR 2. The accuracy of CACS_VNI_ outperformed CACS_VNC_ in comparison with TNC images. However, there was a significant underestimation of CACS_VNI_ values. In another study, Braun et al. [24] scanned 38 patients before transcatheter aortic valve replacement (TAVR). VNC series exhibited a highly effective subtraction of the iodine attenuation component and had satisfactory noise levels. CACS_VNI_ differed significantly from the values measured on TNC scans but showed excellent correlation and outperformed the conventional VNC algorithm. Sharma et al. [25] scanned 88 patients and compared CACS_VNI_, CACS_VNC_, and CACS_TNC_. VNC scored lower median CACSs and VNI scored higher median CACSs compared to TNC. VNI reconstructions outperformed VNC reconstructions regarding the accuracy of CAC quantification and classification. Haag et al. [26] assessed the difference between CACS_VNI_ and CACS_TNC_ among 170 patients. There was no evidence of a difference in the median CACSs, and an excellent correlation was observed between CACS_VNI_ and CACS_TNC_ values, although VNI overestimated the CACS (mean bias of −4.0), and the equivalence test was inconclusive. There was no evidence of a difference between the plaque burden classification (P classification), except that patients without plaque burden were misclassified at higher‐than‐normal rates. Vecsey‐Nagy et al. [27] examined the predictors of CACS_VNI_ calculation accuracy. 197 patients were scanned with either 120 or 140 kVp. The agreement between TNC and VNI was substantial (mean bias of 6.6), but 18.3% of the patients were falsely reclassified as CACS = 0 on VNI. 140 kVp and higher CAC density were the independent predictors of a smaller difference. The studies comparing VNC, VNI, and TNC‐derived CACS values are shown in Table 2. An example of TNC and VNC reconstructions is presented in Figure 2.

**TABLE 2:** Studies comparing VNC, VNI, and TNC‐derived CACS values.

Study	Population	Reconstruction	Results
Emrich et al. [23]	Phantom and 67 patients	70 keV, QIR 2, 3 mm slice thickness	CACS_VNI_ outperformed CACS_VNC_ VNI underestimated CACS
Braun et al. [24]	38 patients	TNC: 70 keV, Qr36, QIR off, 3 mm slice thickness VNC/VNI: Qr36/Br36, QIR 3/4, 0.4/1 mm slice thickness	
Sharma et al. [25]	88 patients	70 keV, Qr36, QIR off, 3 mm slice thickness	CACS_VNI_ outperformed CACS_VNC_ VNI overestimated CACS
Haag et al. [26]	170 patients	TNC: 70 keV, Qr36, QIR off, 3 mm slice thickness VNI: Qr40, QIR 3, 2 mm slice thickness	Strong correlation and limited agreement between CACS_VNI_ and CACS_TNC_ VNI overestimated CACS
Vecsey‐Nagy et al. [27]	197 patients (51 with 120 kVp, 146 with 140 kVp)	70 keV, Qr36, QIR off, 3 mm slice thickness	VNI underestimated CACS 140 kVp and higher CAC density were the predictors of accuracy

Abbreviations: CACS, coronary artery calcium score; keV, kiloelectronvolts; kVp, peak tube voltage in kV; QIR, quantum iterative reconstruction; TNC, true non‐contrast; VNC/VNI, virtual non‐contrast/non‐iodine.

In their phantom and in vivo study, Fink et al. [28] evaluated the impact of VMI and QIR levels on the accuracy of CAC scoring using VNI reconstruction. Phantom and patient data were reconstructed at different VMI and QIR levels, and TNC scans (70 keV, QIR off) were used as reference. CACS_VNI_ and CACS_TNC_ showed an excellent correlation and agreement on all keV and QIR levels. CACS_VNI_ was most accurate compared to CACS_TNC_ at 55 and 60 keV, the effects of QIR level were inconsistent. However, there was an underestimation of CACS at small and low‐density plaques. To address this, in their next study Fink et al. [29] evaluated the impact of plaque size and density on CACS_VNI_ at different VMI and QIR levels in 63 patients. They aimed to determine a modified reconstruction setting for improved detection of small and low‐density plaques to preserve the detectability that would otherwise be false negative. VNI reconstructions were made at different VMI and QIR levels, using a thinner section thickness and increment (1.0 and 1.0 mm) and/or sharper kernel (Qr44). Improved detectability (by 89%) and highest correlation with CACS_TNC_ of small and/or low‐density calcifications at 55 keV, QIR 2 with a 110‐HU threshold was confirmed. To assess the impact of tube current reduction Fink et al. [30] scanned phantoms in varied sizes. VNI reconstructions at 55 keV, QIR1, and 60 keV, QIR 4 showed comparable or even better IQ in terms of noise than TNC reference. CACS_VNI_ at every dose level showed excellent correlation and agreement with CACS_TNC_ at standard radiation dose, with a clinically nonrelevant deviation of scores. CACS_VNI_ at 60 keV, QIR 4 seems to slightly outperform 55 keV, QIR 1 in terms of IQ and score deviation. The optimal VMI reconstruction parameters of VNI images are summarized in Table 3.

**TABLE 3:** Studies regarding optimal VMI reconstruction parameters of VNI images.

Study	Population	Reconstruction	Main result
Fink et al. [28]	Phantom + 61 patients	TNC: 70 keV, QIR off VNI: 55–80 keV, QIR 1–4 both Qr36, 3 mm slice thickness	60 keV, QIR 1 reconstruction had the highest accuracy in patients
Fink et al. [29]	Phantom + 63 patients	TNC: 70 keV, QIR off, Qr36, 3 mm slice thickness VNI: 55–80 keV, QIR 1–4, Qr36 and Qr44, 1 and 3 mm slice thickness	55 keV, Qr44, QIR 2, 1 mm reconstruction with 110 HU threshold had the highest calcification detectability
Fink et al. [30]	Phantom	TNC: 70 keV, QIR off VNI: 55 keV QIR 1, 60 keV, QIR 4 both Qr36, 3 mm slice thickness	60 keV, QIR 4 reconstruction had the best image quality using lower tube current

Abbreviations: HU, Hounsfield unit; keV, kiloelectronvolts; QIR, quantum iterative reconstruction; TNC, true non‐contrast; VNI, virtual non‐iodine.

Fink et al. [31] investigated the impact of cardiac motion and in‐vessel attenuation on CAC scoring in a phantom experiment. Calcium mass, Agatston scores, and cardiac motion susceptibility indices were compared to physical mass and static scores. Their study found that CACS_VNI_ decreased with rising heart rate, but VNI‐based calcium mass and CACS were similar to static values. The standard deviation of cardiac motion susceptibility indices was lower for VNI than for VNC, as well as CACS underestimation, outperforming VNC‐based CAC scoring. In another dynamic phantom study, Dobrolinska et al. [32] showed that reconstruction with QIR off, Qr44 kernel, and 0.4 mm slice thickness improved assessment at all heart rates and calcification densities. Risch et al. [33] revealed no significant heart rate dependence of CACS_VNI_ scanning a dynamic phantom, only increased deviation at higher rates.

VNC and VNI algorithms may also assist in quantifying aortic valve and mitral annular calcifications (AVC and MAC). Mergen et al. [34] assessed the accuracy of VNI‐based AVC, MAC, and CAC measurements on late‐phase scans, compared with TNC images in 99 patients. Based on their outcomes, VNI images enable patient risk stratification and accurate quantification of AVC and MAC in addition to CAC. The best results were achieved at 80 keV, QIR 4 for AVC and MAC, and 70 keV, QIR 4 for CAC. Risch et al. [35] compared the AVC determination of VNC, VNI, and TNC reconstructions on TAVR planning scans of 41 patients. AVC scoring on both series showed near‐perfect correlation but with significant underestimation. VNI with 0.4 mm slices and Br36 kernel at QIR 4 gave the most comparable results. Feldle et al. [36] also found excellent diagnostic performance of VNI reconstruction assessing AVC.

Spectral data‐based VMI image reconstructions enhance image contrast and reduce artifacts. The most accurate reconstruction for CAC scoring was 70 keV, QIR off with 3 mm slice thickness [14]. Optimal VMI reconstructions at lower tube voltages [15, 18, 19], and VMI level–specific CAC thresholds at lower tube current [20] were accurate in CAC scoring compared to standard acquisition in phantom studies.

In determining the CACS from contrast‐enhanced scans, PureCalcium‐derived VNI reconstruction outperformed conventional VNC images and demonstrated an excellent correlation with the CACS_TNC_ reference. However, the accuracy varied between studies [23, 24, 25, 26, 27] and was compromised for calcified lesions of lower density. 140 kVp and higher CAC density were the predictors of a smaller difference [27].

Reconstructions of VNI images at 55 and 60 keV were the most accurate in CACS estimation compared to CACS_TNC_ [28]; 55 keV, Qr44, QIR 2 or less, 1.0 mm slice thickness reconstruction with 120 and 110 HU thresholds were the optimal to detect low‐density calcifications [29]; 60 keV, QIR 4 reconstruction provided the best IQ at lower tube current [30]. CACS_VNI_ showed no significant HR dependency [31, 32, 33]. VNI reconstruction enables accurate quantification of aortic valve and mitral annular calcium [34, 35, 36].

## 3 Coronary CT Angiography (CCTA)

CCTA is the first‐line diagnostic tool in low or moderate (>5%–50%) pre‐test likelihood of obstructive coronary artery disease [37]. PCD‐CT demonstrated improved IQ and diagnostic confidence compared with EID‐CT in CCTA [38]. Spectral data‐based VMI reconstructions can be used to improve CNR, reduce artifacts, and improve the accuracy of stenosis evaluation. Using material decomposition, the virtual removal of calcium aids the stenosis assessment in the case of calcified plaques.

### 3.1 VMIs in CCTA

**FIGURE 3:**
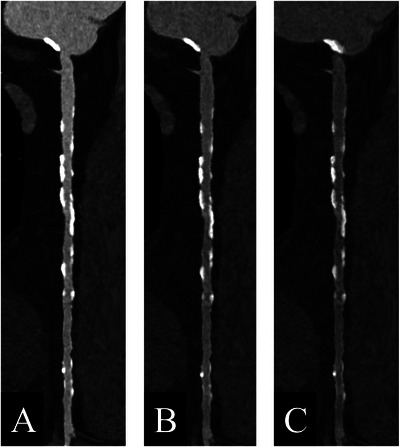
Comparison of different VMI reconstructions. CCTA scan (Siemens NAEOTOM Alpha) of a 67‐year‐old female patient, straight multiplanar view of the right coronary artery. A, B, and C show VMI reconstructions at 70, 90, and 120 keV, respectively, with the same window width and level, both reconstructed with Bv56 kernel, QIR 3, and 0.4 mm slice thickness.

**FIGURE 4:**
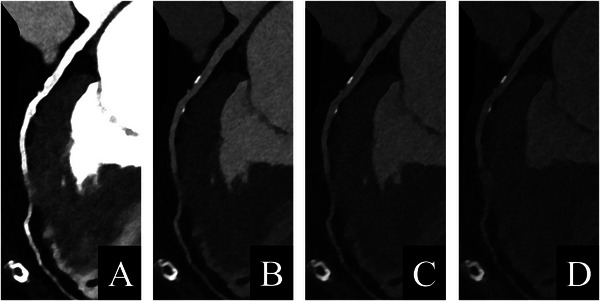
Comparison of different VMI reconstructions. CCTA scan (Siemens NAEOTOM Alpha) of a 72‐year‐old female patient, curved multiplanar view of the left anterior descending artery. A, B, C, and D show VMI reconstructions at 40, 70, 90, and 120 keV, respectively, with the same window width and level, both reconstructed with Bv40 kernel, QIR 3, and 0.4 mm slice thickness.

Greffier et al. [39] showed that PCD‐CT images gave greater detectability of the coronary lumen for 40–90 keV VMIs compared to two EID‐DECT systems, with the benefits of higher lumen sharpness and overall quality. Sartoretti et al. [40] assessed systematically the impact of keV and QIR levels on CCTA images. They scanned (prospective ECG‐triggered, 120 kVp, automatic tube current modulation) a phantom with dynamic flow, pulsatile heart motion, and different calcified plaques with various stenosis grades; 10 patients underwent CCTA. VMI reconstructions were made at 40–70 keV with QIR off and all levels (1–4), with Bv40 kernel and 0.6 mm slice thickness. Considering all data, 40 keV QIR 4 reconstruction offered optimal objective and subjective IQ. Wolf et al. [41] studied the impact of keV levels on quantitative stenosis assessment. They scanned a dynamic phantom with artificial vessels and 33 patients who underwent invasive coronary angiography (ICA) after CCTA. For calcified and mixed plaque, percentage diameter stenosis and calcium blooming both decreased with increasing VMI reconstruction levels. Owaga et al. [42] investigated the effect of heart rate and VMI levels on coronary stent imaging. A dynamic phantom with five types of stents and two stents with 50% in‐stent restenosis was scanned. A measurable lumen, even at high heart rates, was maintained. For in‐stent stenosis assessment, the residual lumen was larger, the CNR was higher, and the IQ was better at lower keV levels (40 keV). Liu et al. [43] scanned a dynamic coronary stenosis phantom at different heart. Different VMI reconstructions and iodine density images were analyzed. Heart rate and acquisition mode did not affect spectral results. VMI reconstructions of CCTA scans are presented in Figures 3 and 4.

VMI reconstruction may help to reduce contrast media (CM) volume but maintain high IQ. In the study of Emrich et al. [44], a dynamic phantom was scanned using a clinical contrast injection protocol with standard (100%) and reduced contrast agent concentrations (75%–20%). VMI reconstruction at 40–70 keV and a polychromatic reference were made. Results showed that contrast media concentration could be reduced by 50% to obtain diagnostic attenuation at 40 keV. In the study of Rajiah et al. [45], patients were administered either low (30 mL) or normal routine (60 mL) volumes of contrast, and they were scanned with high‐pitch helical, multi‐energy mode. The 50 keV VMI was superior to polychromatic images with higher vascular enhancement and CNR. Thus, 50 keV VMIs may be able to salvage non‐diagnostic contrast‐enhanced studies. Cundari et al. [46] determined that the best VMI level regarding objective and subjective IQ for CCTA is 45 keV. Two patient groups were scanned with 20% and 40% contrast media reduction; the vascular attenuation and CNR were compared using 45 keV VMI reconstructions. They found that a diagnostic IQ can be maintained by exploiting the lower noise and higher CNR properties of low‐energy‐level VMI reconstructions. The studies regarding VMI reconstruction of CCTA images to improve IQ and save contrast media are summarized in Table 4.

**TABLE 4:** Studies regarding VMI reconstruction of CCTA images to improve image quality and save contrast media.

Study	Population, design	Measurements	Results
Greffier et al. [39]	Phantom, EID‐CT ref.	Objective and subjective image quality analysis	PCD‐CT outperformed EID‐CT
Sartoretti et al. [40]	Dynamic phantom 10 patients	Objective and subjective image quality analysis	40 keV QIR 4 reconstruction had the optimal image quality
Wolf et al. [41]	Dynamic phantom 33 patients, ICA ref.	Quantitative stenosis assessment	Lowest PDS bias: ‐ for calcified plaque at 100 keV ‐ for mixed plaque at 140 keV ‐ for non‐calcified plaque at 40 keV lowest blooming at 140 keV
Owaga et al. [42]	Dynamic phantom with stents and ISR	Lumen measurement; objective and subjective image quality analysis	Lumen diameter was heart rate independent residual lumen was more visible at 40 keV 40 keV reconstruction had the best image quality
Liu et al. [43]	Dynamic phantom	Quantitative metrics	No effect of heart rate, keV level (50–150) or acquisition type (sequential, helical, high‐pitch helical)
Emrich et al. [44]	Dynamic phantom	Image quality analysis	40 keV reconstruction obtained diagnostic attenuation with 50% contrast media concentration reduction
Rajiah et al. [45]	53 patients randomized (1:1) to full and half CM dose	Objective and subjective image quality analysis	50 keV reconstruction had higher vascular enhancement and CNR, CM dose can be reduced
Cundari et al. [46]	100 patients (1:1:1) full, 80% and 60% CM dose	Objective and subjective image quality analysis	45 keV reconstruction had the best image quality, CM dose can be reduced

Abbreviations: CM, contrast media; CNR, contrast‐to‐noise ratio; EID‐CT, energy‐integrating detector CT; ICA, invasive coronary angiography; ISR, in‐stent restenosis; keV, kiloelectronvolts; PCD‐CT, photon‐counting detector CT; PDS, percentage diameter stenosis.

### 3.2 Lumen and Stenosis Assessment With Material Decomposition

Severely calcified plaques with blooming artifacts negatively affect the diagnostic performance of CCTA [47]. VNCa images can be reconstructed based on spectral data with dedicated algorithms (e.g., PureLumen), applying material decomposition. This method may enable accurate stenosis measurement in vessels with extensive calcification.

**FIGURE 5:**
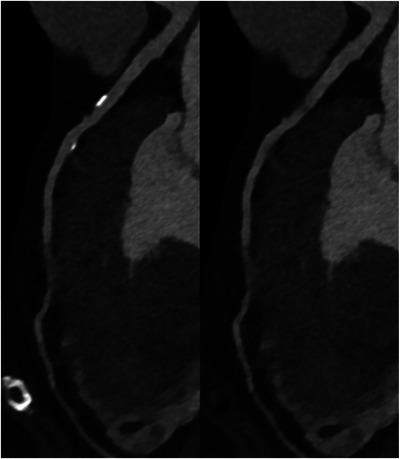
VNCa algorithm‐based calcium removal. Left anterior descending artery of a 72‐year‐old female patient. CCTA scan (Siemens NAEOTOM Alpha), reconstructed at 70 keV with Bv40 kernel, QIR 3, and 0.4 mm slice thickness.

Allmendinger et al. [48] scanned a dynamic phantom with two contrast‐filled vessels with 25% and 50% stenoses at different simulated heart rates. VMI and VNCa reconstructions were made at 65 keV with identical reconstruction parameters. Results showed that VNCa‐derived stenosis measurements were closer to the actual stenosis. A visual assessment of the IQ depending on the heart rate yielded good IQ up to a heart rate of 80 bpm. In another dynamic phantom study, Zsarnoczay et al. [49] compared ultra‐high resolution (UHR), standard resolution, and VNCa reconstructions in coronary artery stenosis evaluation. Both the UHR and the VNCa techniques provided improved quantification compared to standard resolution. VNCa maintained its performance for the 50% stenosis from lowest to highest heart rates, for the 25% stenosis, VNCa maintained its performance until 80 bpm, and UHR was heart rate independent. In the first in vivo study, Mergen et al. [50] compared diameter stenosis quantified on VMI and VNCa images with 3D‐QCA (quantitative coronary angiography) as reference. 10 of the 81 plaques were excluded due to erroneous plaque subtraction on VNCa images. As compared with 3D‐QCA, VNCa images showed similar diameter stenoses, therefore suggesting its potential to improve the quantification of calcified stenoses. VNCa algorithm‐based calcium removal is presented in Figure 5.

VMIs can be generated based on spectral information to improve CNR and reduce artifacts. To achieve the optimal IQ [40] and visualize low attenuation in‐stent restenosis [42], lower (40 keV) VMI levels are recommended. For accurate evaluation of stenotic calcified plaques and less blooming artifacts, higher (90–100 keV) VMI levels are recommended [41]. The IQ of VMI reconstructions is heart rate independent [42, 43]. With low (40–50 keV) VMI level reconstructions, IQ can be diagnostic with up to 50% contrast media reduction [44, 45, 46].

VNCa images can be reconstructed to eliminate calcium‐related blooming artifacts. In dynamic phantom studies, VNCa images provided accurate stenosis measurements up to 80 bpm in phantom studies [48, 49]. In a clinical study, VNCa‐derived stenosis quantification showed similar values compared with QCA results [50]. However, discrepancies have emerged among studies regarding the selection of the optimal kernel and reconstruction parameters. Future studies need to focus on the systematic evaluation of the data and optimization. On the current clinically available PCD‐CT, spectral data collection has become possible in UHR mode, which will contribute to a direct in vivo comparison of UHR and VNCa stenosis quantification.

## 4 Myocardium Characterization

### 4.1 Myocardial Perfusion

Although CCTA enables ruling out obstructive CAD, the morphologic information provided remains insufficient to determine the downstream functional impact of coronary plaques. Myocardial perfusion imaging (MPI) is mostly focused on ischemia or infarction detection and evaluating myocardial viability. Spectral data are applicable to calculate iodine concentration (see in detail below), and VMIs can also be created to improve IQ [51, 52]. In a phantom study, iodine quantification using PCD‐CT was accurate compared to EID‐based dual‐source, dual‐energy scanners [53]. The spectral capabilities of PCD‐CT in MPI have not been investigated yet.

### 4.2 Myocardial Extracellular Volume (ECV)

The myocardial ECV is the proportion of extracellular matrix of the whole myocardium. High ECV indicates interstitial fibrosis, edema, or infiltration (e.g., amyloidosis). Cardiac magnetic resonance imaging (CMR) is the gold standard method for non‐invasive ECV quantification. ECV quantification with CT requires a late enhancement (LE) scan acquired at least 5 min after contrast media injection to reach relative iodine equilibrium. CT‐based ECV can be calculated with two different methods: (1) the subtraction or single‐energy (SE) method, which uses the attenuation (HU) differences between pre‐contrast and LE images; and (2) the iodine density or dual‐energy (DE) method, which requires spectral information. Iodine concentration can be calculated based on material decomposition, and a color‐coded map can be generated to visualize iodine distribution. The myocardium‐to‐left ventricle iodine density ratio is used to determine the myocardial ECV. Both methods need recent hematocrit values for the estimation (see formula below). The iodine density method yielded more accuracy than the subtraction method and was comparable with CMR‐derived ECV in dual‐layer dual‐energy CT studies [54, 55, 56, 57].

(1) $$\begin{array}{c} ECV\left(\mathrm{SE}\right)=\left(1-hematocrit\right)\times \Delta attenuation_{\mathrm{myo}}/\Delta attenuation_{\mathrm{blood}} \end{array}$$

(2) $$ECV\left(\mathrm{DE}\right)=\left(1-hematocrit\right)\times \frac{\left[iodine_{\mathrm{myo}}\right]}{\left[iodine_{\mathrm{blood}}\right]}$$

**FIGURE 6:**
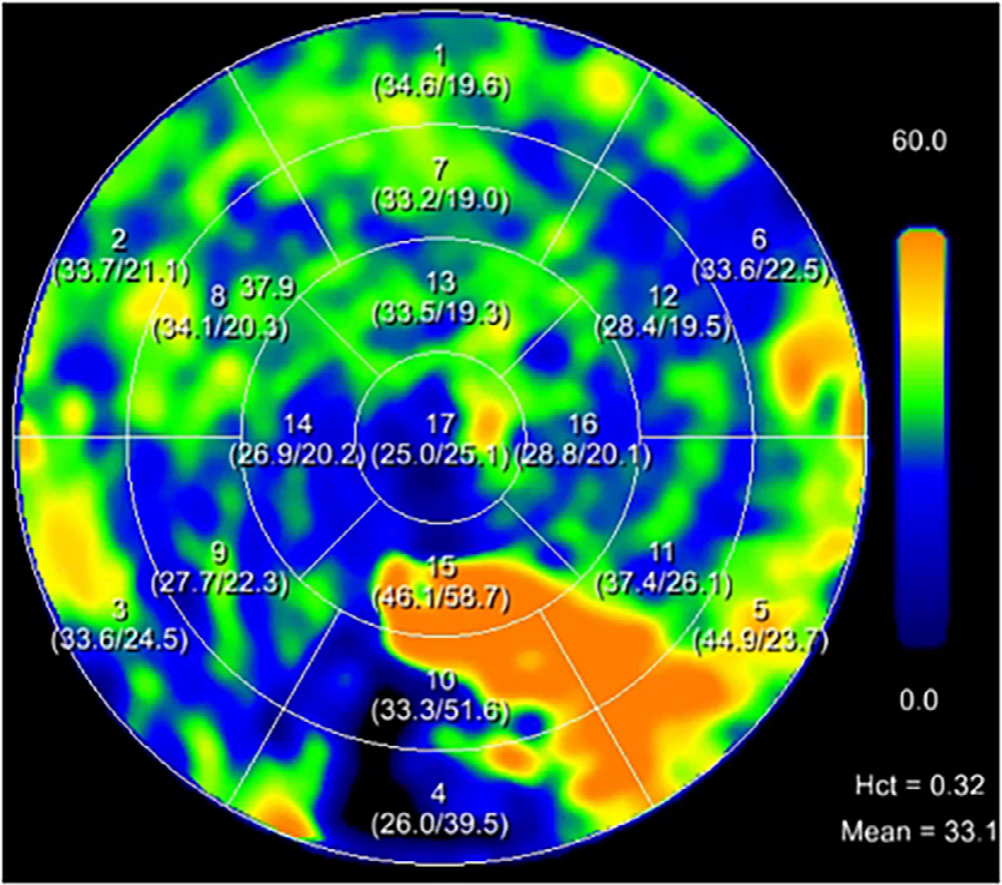
ECV quantification. Color‐coded (polar) map of ECV quantification results. The numbered areas (1–17) represent myocardial segments according to the 17‐segment model. Each segment is annotated with the segmental ECV values. The heatmap uses a color scale (blue to orange) to represent ECV percentages, with blue indicating lower ECV values and orange indicating higher ECV values. This example shows increased inferolateral ECV percentages.

Mergen et al. [58] studied the feasibility and accuracy of spectral data‐based ECV quantification using PCD‐CT. 30 patients with severe aortic stenosis were scanned before TAVR. LE cardiac scan was acquired 5 min after the contrast injection as part of the protocol. VMI images at 65 keV, QIR 3 were reconstructed for the unenhanced, CCTA and LE scans, and iodine images were generated from the LE scan. ECV was calculated in both ways with commercially available dedicated research software. The ECV quantification with the two methods showed a high correlation between each other with small mean errors and narrow limits of agreement; therefore, LE scan obviates the need for a non‐enhanced scan, which leads to radiation dose reduction. In the prospective study of Aquino et al. [59], 29 patients with several different indications underwent both PCD‐CT and CMR examination on the same day. The unenhanced and LE CT protocol and the image reconstruction parameters were identical to the previous study. The CMR protocol included myocardial native and postcontrast T1 mapping and late gadolinium enhancement imaging. Global and midventricular ECV were measured in three ways: subtraction and iodine density method from CT images, and T1 mapping method from CMR images. There was a strong correlation between the two CT methods for ECV quantification, with 40% less radiation dose using iodine mapping. In comparison with MRI, the iodine mapping method showed a strong correlation and good to excellent reliability for midventricular and global ECV quantification, but it overestimated ECV by approximately 2% (subtraction method overestimated by 3%). ECV quantification is presented in Figure 6.

Gnasso et al. [60] evaluated the impact of reconstruction settings on the accuracy of ECV quantification (compared to CMR). The most optimal reconstruction setting for the LE scans was 45 keV, QIR 4 with 0.4 mm slice thickness. 63% reduction in mean bias and a 6% increase in concordance with MRI‐based ECV were achieved compared to standard settings (65 keV, QIR 3 with 1.5 mm).

Hematocrit is essential in myocardial ECV calculation (based on CT and CMR images as well). Attenuation of the blood pool determined on VNC images allows quantification of hemoglobin level [61, 62]. In a prospective study, Mergen et al. [63] assessed the accuracy of ECV measurement based on synthetic hematocrit from VNC and VNI images. 75 patients were used as a derivation cohort, and 50 patients served as a validation cohort who underwent CT scans before TAVR. Myocardial ECV values were similar between methods; therefore, synthetic hematocrit calculation from VNC images enables an accurate computation of myocardial ECV.

To determine myocardial ECV, the subtraction method and the spectral data‐based iodine density method can be used. Studies showed a strong correlation between the two methods. In comparison with the CMR reference, the iodine mapping method showed stronger correlation and better reliability than the subtraction method, with 40% less radiation dose, but approximately 2% overestimation [59]. The optimal LE scan reconstruction parameters were 45 keV, QIR 4 with 0.4 mm slice thickness [60]. Further research is needed to optimize the LE phase time. Synthetic hematocrit calculated from VNC images enables an accurate computation of myocardial ECV [63].

## 5 Other Biomarkers

### 5.1 Epicardial Adipose Tissue (EAT)

EAT attenuation, EAT volume, and fat attenuation index (FAI) are biomarkers of perivascular inflammation and an indicator of increased cardiac mortality [64]. VMI reconstruction energy level may affect the measurement of EAT and FAI by changing HU values.

Mergen et al. [65] assessed EAT attenuation and FAI at different monoenergetic levels. An anthropomorphic phantom was scanned with PCD‐CT and EID‐CT as a reference, and 30 patients underwent calcium scoring and CCTA. EAT attenuation and FAI were impacted by keV level and contrast enhancement. VMI reconstruction at 70 keV provided fat attenuation approximating conventional polychromatic measurements. Furthermore, an ex vivo porcine heart study obtained equivalent results [66]. EAT volume can also be measured on non‐enhanced and contrast‐enhanced images, with different HU thresholds. Risch et al. [67] investigated the influence of calcium removal algorithms on EAT volume and attenuation measurement. 42 patients underwent unenhanced scans and CCTA. VNI, VNC, and TNC images were reconstructed at 70 keV, and measurements were made on the CTA series as well, with two HU thresholds. VNI showed superior and more consistent results for EAT volume, and the best agreement in the distribution of EAT attenuation values compared to the TNC reference. In the study of Cui et al. [68], VNI images provided a more accurate EAT volume and density measurement and radiomic feature reproducibility than VNC images compared to TNC.

### 5.2 Plaque Component Volumes

Quantifying plaque morphological characteristics and plaque burden, in addition to stenosis assessment, can improve cardiovascular risk prediction since total plaque and low‐attenuation plaque quantities are associated with adverse events [69, 70]. VMI reconstructions change the HU values of the voxels and, therefore, may impact plaque volume estimation. Vattay et al. [71] analyzed 51 plaques from 51 patients to assess how plaque component volumes change with keV level. Quantitative plaque analysis was performed on polychromatic (T3D) reference images, and segmentation masks were copied to VMI reconstructions. Average attenuation and CNR decreased significantly with increasing keV levels, with similar values observed between T3D and 70 keV images. Non‐calcified plaque volume was comparable between T3D and 100–180‐keV reconstructions. There was a monotonic decrease in mean calcified plaque volume, with a significant difference between all VMIs and T3D. Low attenuation plaque volume increased with increasing keV levels, and all VMIs showed a significant difference compared to T3D, except for 50 keV.

## 6 Conclusion

PCD‐CT technology offers higher CNR and better spatial resolution compared to EID‐CT, also providing detector‐based spectral information. PCD‐derived material decomposition and VMI reconstructions enable accurate assessment of cardiac diseases compared to gold standard imaging, based on the initial studies. VMI reconstructions offer improved IQ, therefore are generally used in the daily clinical practice (e.g., 70 keV VMI for calcium scoring). VMI can be used for radiation dose or contrast media reduction and can salvage suboptimal data due to low contrast media volume. In terms of material decomposition‐based algorithms (VNC, VNI, VNCa), recent studies show promising results on iodine removal to reduce radiation dose by avoiding unenhanced scans and improve stenosis assessment by eliminating calcium‐related blooming artifacts. In the evaluation of the myocardium, the role of cardiac CT examination can be extended. Late phase scans can be added to CCTA scans to characterize the myocardium and obtain additional information regarding ECV. The significance of epicardial and pericoronary adipose tissue attenuation in risk prediction is promising; however, it needs further evaluation since spectral reconstructions may significantly influence attenuation‐based data.

Further research with larger cohorts and clinical trials is needed to confirm previous findings, determine optimal acquisition and reconstruction parameters for different situations, provide longitudinal data on patient care, and implement the results into clinical guidelines.

### 6.1 Possible Future Developments in Clinical Practice

Improved calcium removal algorithms will eliminate the need for TNC scans; therefore, the implementation of this method will reduce scan time and total radiation dose. The optimization of high‐quality PCD‐CT images with VMI reconstructions will allow further contrast media and radiation dose reduction. The further development of the calcium removal algorithms will aid in the measurement of stenosis caused by extensive calcified plaques. Myocardial ECV measurement using spectral data will provide additional information regarding the myocardium; thus, in some cases, CMR will be redundant.
